# Altered multisensory integration in pilots: Examining susceptibility to fission and fusion sound-induced flash illusions

**DOI:** 10.1177/20416695251364202

**Published:** 2025-08-06

**Authors:** Xing Peng, Yaowei Liang, Xiuyi Li, Jiaying Sun, Xiaoyu Tang, Aijun Wang, Chengyi Zeng

**Affiliations:** 1 117786 Civil Aviation Flight University of China, China; 2 7938 University of Toronto, Canada; 3 66523 Liaoning Normal University, China; 4 12582 Soochow University, China

**Keywords:** sound-induced flash illusions, multisensory integration, temporal binding windows, pilots, signal detection theory

## Abstract

Pilots show superior visual processing capabilities in many visual domain tasks. However, the extent to which this perceptual advantage extends to multisensory processing requires validation. In this study, we examined multisensory integration of auditory and visual information in both pilot and control groups, utilizing two sound-induced flash illusions (SIFI) tasks: the fission illusion, where one flash coupled with two beeps is perceived as two flashes; and the fusion illusion, where two flashes with a single beep are perceived as one flash. Sixty-six participants were instructed to discern whether they observed one or two flashes while discounting irrelevant auditory beeps, across six conditions: one flash (1F), two flashes (2F), one flash/one beep (1F1B), one flash/two beeps (1F2B), two flashes/one beep (2F1B), and two flashes/two beeps (2F2B). We varied six stimulus onset asynchronies (SOAs) between auditory and visual events (25–150 ms) to assess the participants’ temporal binding window (TBW). Signal detection theory was employed to analyze the group differences in illusion reports. The findings suggest that, while pilots are less susceptible to SIFI in either fission or fusion conditions, they only exhibit narrower TBW in the fusion condition, where pilots demonstrated a more gradual change in their susceptibility as SOA increases. In the fission condition, the group difference was primarily driven by visual sensitivity, whereas in the fusion condition it also likely reflected pilots’ distinct multisensory integration mechanisms. Two alternative possibilities are discussed to explain the group differences and the different multisensory integration patterns in fission and fusion conditions.

## How to cite this article

Peng X, Liang Y, Li X, Sun J, Tang X, Wang A, & Zeng C. (2025). Altered multisensory integration in pilots: Examining susceptibility to fission and fusion sound-induced flash illusions. *i-Perception*, *16*(4), 1–18. https://doi.org/10.1177/20416695251364202

## Introduction

Individuals are continuously exposed to varying sensory information, including visual, auditory, and tactile stimuli, along with their combinations, collectively referred to as multisensory stimuli. Such stimuli are prevalent in high-demand operational areas such as healthcare, transportation, and aviation, where they are recognized for enhancing detection, improving the accuracy of localization, and eliciting faster reactions (Marucci et al., 2021; Sangari et al., 2023; Spence & Ho, 2008; Wickens et al., 2008). Beyond these benefits, multisensory stimuli will also cause perceptual illusions. A notable example is the Sound-Induced Flash Illusion (SIFI), suggested by Shams et al. (2000, 2002). In this illusion, the presence of two auditory stimuli with one visual stimulus (1F2B) can create the perception of an additional visual event, known as the fission illusion, whereas one auditory stimulus paired with two visual stimuli (2F1B) can lead to the perception of a single visual event, referred to as the fusion illusion (Andersen et al., 2004; Shams et al., 2000, 2002).

Research has shown that the SIFI is highly dependent on the temporal characteristics of the stimuli, particularly their level of synchrony. It has been suggested that multisensory stimuli presented in close temporal proximity may be integrated into a single, unified percept. This integration tends to easily occur within the brain's temporal window, known as the multisensory temporal binding window (TBW) (Colonius & Diederich, 2004; Powers et al., 2009), while outside the TBW, the possibility of integration will decrease significantly.

Correspondingly, the closer the temporal proximity between two stimuli, the more likely they are to be integrated into a single percept. However, as the temporal interval between stimuli—specifically, the stimulus onset asynchrony (SOA)—increases, the likelihood of multisensory integration decreases. Auditory and visual stimuli are typically integrated into a single perceptual representation at SOA ranging from physical simultaneity (0 ms apart) to approximately 100–150 ms, beyond which the stimuli are perceived as separate events (Schneider & Bavelier, 2003; Zampini et al., 2005). Additionally, the TBW is asymmetric: visual-leading pairs (vision first, sound second) remain perceptually integrated over a wider TBW than auditory-leading pairs, aligned with an ecological validity of seeing before hearing in natural settings (Vroomen & Keetels, 2010).

Individual differences have also led to inconsistent results in terms of susceptibility to SIFI that both narrower and broader TBWs have been observed in some populations, including those with autism spectrum disorders (Bao et al., 2017; Foss-Feig et al., 2010), amblyopia (Narinesingh et al., 2017), synesthesia (Neufeld et al., 2012), schizophrenia (Haß et al., 2017), and aging (Innes-Brown et al., 2011; McGovern et al., 2014). Additionally, specific long-term perceptual-cognitive experiences have been shown to correlate with the length of the TBW. For instance, individuals with specialized auditory experiences, such as musicians and bilinguals, have demonstrated both faster and more accurate processing of concurrent audiovisual cues, with stronger activity in the frontal cortex (BA 10) associated with faster perceptual response, and with less illusion-induced changes in event-related potentials (ERP) latencies at central scalp sites (electrodes C1, Cz, C2); non-musicians and monolinguals exhibited greater susceptibility to audiovisual illusions, perceiving double flashes across a wider range of SOAs (Bidelman, 2016; Bidelman & Heath, 2019). Similarly, individuals with significant audiovisual or visually dominated experiences, such as video game players, have shown narrower TBW compared to non-players or those with less experience (Di Luzio et al., 2021; Donohue et al., 2010). Furthermore, even within the same perceptual environment, a recent study revealed that professional goalkeepers possess narrower TBW and a predisposition to segregate sensory signals compared to professional outfield players and control participants (Quinn et al., 2023). This highlights the influence of specific demands to make rapid decisions based on the same audiovisual cues.

This study expands the scope of the topic of group differences in SIFI by investigating the pilots’ proneness to SIFI and their TBW. Similar to previous reports on special groups, we assume that pilots will process multisensory stimuli differently compared to the control group because there are high demands for them to deal with rich visual and auditory information, such as complex interface parameters and immersive radio communications, and requirements to quickly and accurately discriminate between multimodal warning signals which are directly related to critical issues (Dehais et al., 2018; Dehais et al., 2019).

Studies have shown that pilots have superior visual processing capabilities (Sterkin et al., 2018), including efficient attention allocation (Shao et al., 2021; Yu et al., 2016), visual working memory (Liang et al., 2024), and visuospatial orientation (Dror et al., 1993; Ledegang & Groen, 2018). Our previous study also showed that pilots may benefit from capturing attention from multisensory warning signals in different SOA (Peng et al., 2022); however, this potential different multisensory processing mechanism reflected in SIFI is still unclear.

The current study investigated the pilots’ proneness to SIFI and their TBW. To do this, we conducted experiments in which pilots and non-pilots were exposed to the SIFI paradigms, specifically the fission (1F2B) and fusion (2F1B) conditions. We systematically varied the SOA from 25 to 150 ms for beeps and flashes to evaluate the TBW (Shams et al., 2000, 2002), and we used Signal Detection Theory (SDT) to analyze if changes in the proneness to illusions were due to shifts in perceptual sensitivity *d’* or response criterion *ln(β)* (Watkins et al., 2006; Whittingham et al., 2014). We hypothesize that pilots will process audiovisual cues more accurately than their non-pilot counterparts and will demonstrate more refined TBW.

## Materials and Methods

### Participants

Sample size calculations were performed in G*Power (Version 3.1) (Faul et al., 2007) for two purposes. First, for a mixed-design ANOVA with two groups (pilot vs. control) and six SOAs (25, 50, 75, 100, 125 and 150 ms), with effect size *f* = 0.25, α = .05, power (1 − β) = 0.9, the minimum required *N* was 24. Second, for an independent-samples *t*-test comparing TBW between groups, with effect size *d* = 0.9, α = .05, power (1 − β) = 0.9, the required *N* was 54.

Sixty-six participants agreed to take part in the study. The control group consisted of thirty-six participants (36 males, 18–24 years, *M* = 21.48 years) who were students at Liaoning Normal University. The pilot group consisted of thirty participants (30 males, 21–23 years, *M* = 22.07 years) recruited from the Civil Aviation Flight University of China. An independent-sample *t*-test confirmed that the two groups did not differ significantly in age, *t*(64) = −1.75, *p* = .10. All pilot participants held commercial aviation licenses from the government administration and had logged an average of over 230 hr of flight time in simulators and real aircraft. All participants are third-year or fourth-year undergraduate students. They have similar second language proficiency as all passed the Chinese College English Test (CET-4). All participants reported <2 hr per week of any kind of video game in the past 6 months, and they reported that they had no professional training in at least one musical instrument. All participants reported normal or corrected-to-normal vision and hearing, with no history of neuropsychiatric illnesses. Prior to the experiment, all participants provided written informed consent. All procedures were conducted in accordance with prescribed ethical standards, and the protocols were approved by the ethics committees of Liaoning Normal University and the Civil Aviation Flight University of China. This research complied with the 1964 Helsinki Declaration.

### Stimuli and Procedure

**Figure 1.**
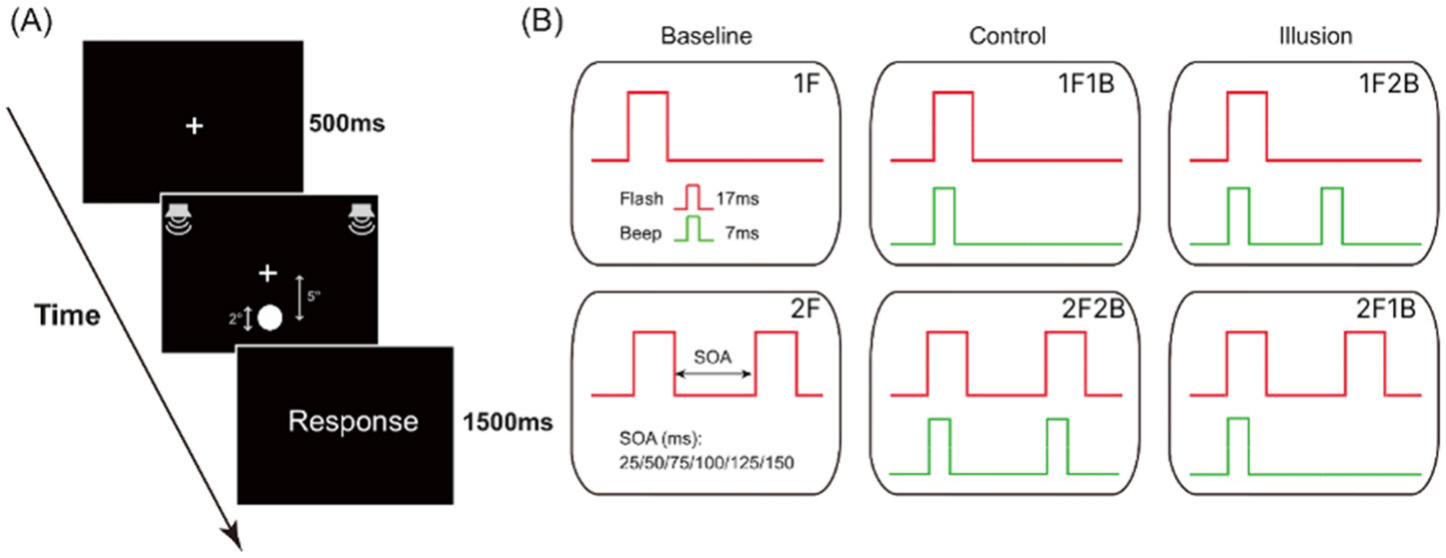
Procedure and temporal profile of stimuli presentation. (A) Schematic of the stimuli procedure. (B) The time course of the presentation in six conditions.

During the experiment, participants were seated approximately 60 cm from the screen in a dedicated, dimly lit, and soundproof room. Stimuli were displayed on a 19.1-in. Lenovo LEN1152 L197 monitor with a refresh rate of 60 Hz and a resolution of 1920 × 1080 pixels. The Presentation software (Neurobehavioral Systems, Inc.) controlled stimuli delivery and response data collection. The visual stimuli consisted of a white disc (2° visual angle) with a luminance of 148 cd/m^2^, presented for 17 ms against a black background in the peripheral visual field (5°) below a centrally presented white fixation cross, which remained on the screen throughout the experiment. The auditory stimuli were 65 dB, 3500 Hz pure tones with a duration of 7 ms (ramped on and off for 3 ms). The inter-trial interval (ITI) is 2000 ms. These were presented in the free field simultaneously from speakers attached to the right and left sides of the monitor display, resulting in centrally localized sounds (Fig. 1A).

Participants encountered one or two visual flashes paired with zero, one, or two auditory beeps per trial, covering all possible flash-beep combinations. The baseline condition included a single flash (1F) and double flash (2F). The control condition included one flash with one beep (1F1B) and two flashes with two beeps (2F2B) pairings. The illusion conditions comprised one flash with two beeps (1F2B), indicative of the fission illusion, and two flashes with one beep (2F1B), indicative of the fusion illusion. In all conditions (i.e., 2F, 2F1B, 1F2B, 2F2B) with two repeated stimuli, the repetitions were separated by a variable SOA (25, 50, 75, 100, 125 and 150 ms). For example, in the 2F2B condition, the first flash–beep pair was presented, and the second flash–beep pair followed after a variable SOA (Fig. 1B). An equal number of trials were conducted with 30 trials per SOA setting. For conditions without SOA setting (i.e., 1F, 1F1B), participants simply repeated 180 trials. Therefore, participants completed overall 1080 trials across six blocks, with the sequence of trials randomized.

In each trial, participants were instructed to fixate on the point and respond to the number of flashes presented by pressing the corresponding key (1 or 2) on the numeric keyboard while ignoring the auditory stimulation. Before the experiment, participants performed a few practice trials to ensure they understood the task. The experiment lasted approximately 1 hr.

### Data Analysis

#### Behavioral Data

We calculated the average percentage of trials in which participants wrongly reported for each SOA. For example, in 1F2B presentations (fission trials), a higher percentage suggests participants mistakenly reported two flashes when only one was shown. In 2F1B presentations (fusion trials), a higher percentage indicates that participants mistakenly reported one flash when two were shown. The mixed ANOVA will be used to analyze the effect of between-subjects factor of group, within-subjects factor of SOAs and their interaction. If the main effect or interaction effect is significant, post hoc pairwise comparisons with Bonferroni correction will be applied to compare each single condition. The adjusted degree of freedom was reported using the Greenhouse–Geisser method when the sphericity assumption was not met.

#### TBW Measurement

We aimed to characterize the TBW of the SIFI by identifying the SOAs that significantly elicit the effect. We used the curve-fitting approach suggested by previous studies (Di Luzio et al., 2021; McGovern et al., 2022; Stevenson & Wallace, 2013; Stevenson et al., 2012; Zerr et al., 2019). Specifically, for each participant, we plotted the proportion of illusion reports (*y*) against SOA (*x*) and fitted a suggested sigmoid function:

$$y=a+\frac{b}{1+e^{\frac{-(x-c)}{d}}}$$

where *a* and *b* are lower and upper asymptotes, *c* is the inflection point, *d* is the slope (Di Luzio et al., 2021; Venskus et al., 2021). Here, the TBW is defined using the inflection point *c*, which precisely marks the SOA where illusion probability begins to significantly decline (Cecere et al., 2015; Di Luzio et al., 2021). We fitted the curve and estimated *c* for each participant in SPSS 29 using the Nonlinear Regression toolbox. The overall goodness of fit to the model was also evaluated for both groups by means of average SSE and *R*-squared values, calculated for every participant. We excluded 3 control participants and 5 pilot participants from the data analysis because their fitted results had an *R²* value lower than 50%. We compared the resulting TBWs between groups using independent-sample *t*-tests.

Compared to the traditional method defining TBW by a fixed *y*-value (McGovern et al., 2022; Stevenson et al., 2012; Zerr et al., 2019), the inflection point method we used could consider more participants who exhibit consistently high or low probabilities of illusions at all SOAs.

#### SDT Analysis

We utilized SDT to determine if changes in the probability of illusions resulted from changes in perceptual sensitivity, the response criterion, or a combination of both, following methodologies from previous studies (Keil, 2020; Vanes et al., 2016). At each SOA (25, 50, 75, 100, 125, and 150 ms), we calculated each participant's visual sensitivity *d’* and the response criterion *ln(β)* respectively. The calculations are as follows:

(1) $$d^{\prime }=z(hit)-z(\;false\;alarm)$$

(2) $$ln(\beta )=\frac{z{\left(\;false\;alarm\right)}^{2}-z{\left(hit\right)}^{2}}{2}$$

Here, “*hit*” denotes the correct identification of the number of flashes at one SOA in the control condition, while “*false alarm*” refers to the incorrect identification of additional flashes at the same SOA in the illusion condition. Because the metrics assess discriminability between one and two flashes, the calculations should control for the auditory-beep by comparing control and illusion conditions (i.e., 2F2B vs. 1F2B; 1F1B vs. 2F1B). Specifically, for fission trials (i.e., 2 beeps), a response of “2” for 2F2B is a “*hit*” and “1” is a “*miss*”; a response of “2” for 1F2B is a “*false alarm*” and “1” is a “*correct rejection*.” The *d’* measures the ability to discriminate between one and two flashes when accompanied by two beeps and negative *ln(β)* indicates a bias towards responding “2.” For fusion trials (i.e., 1 beep), a response of “1” for 1F1B is a “*hit*” and “2” is a “*miss*”; a response of “1” for 2F1B is a “*false alarm*” and “2” is a “*correct rejection*.” Notably, since 1F1B does not involve SOA manipulation, the value of “*hit*” for fusion trials remains the same across SOAs when computing *d’* and *ln(β)* for each individual. The *d’* assesses the ability to discern between one and two flashes in the presence of a single beep, and a negative *ln(β)* implies a bias towards “1.” The z(*p*) is the inverse of the cumulative distribution function of the normal distribution. Incidents of *p* = 0 and *p* = 1 were approximated by 1/2*N* and 1 − (1/2*N*), respectively, where *N* is the number of trials tested (Hautus, 1995; Stanislaw & Todorov, 1999). We used mixed model ANOVA to examine the impact of group and SOA on the probability of illusory responses, perceptual sensitivity *d’*, and response criterion *ln(β)*.

## Result

### Probability of Illusion

First, we compared the performance of pilot and control groups on baseline and control conditions (1F, 1F1B, 2F, 2F2B) across different SOAs, establishing a reference. The independent-sample t-test was used to compare two groups’ error rates in the 1F and 1F1B conditions. There was no significant difference in error rates between the two groups in 1F condition, *t*(64) = 0.42, *p* = .68, either in 1F1B condition, *t*(64) = −0.98, *p* = .33 (Table 1). Pilots and control groups showed similar performance in these two conditions.

**Table 1.** Mean (*SD*) proportion of fission (1F2B) and fusion (2F1B) illusions by group across SOAs.

Condition	Group	Overall	25 ms	50 ms	75 ms	100 ms	125 ms	150 ms
1F	Pilot	0.06 (0.10)						
	Control	0.07 (0.11)						
1F1B	Pilot	0.08 (0.07)						
	Control	0.06 (0.09)						
2F	Pilot	0.26 (0.13)	0.62 (0.32)	0.50 (0.35)	0.20 (0.23)	0.11 (0.13)	0.06 (0.08)	0.07 (0.09)
	Control	0.38 (0.13)	0.94 (0.07)	0.79 (0.23)	0.30 (0.23)	0.10 (0.10)	0.07 (0.10)	0.07 (0.09)
2F2B	Pilot	0.15 (0.25)	0.42 (0.35)	0.28 (0.29)	0.06 (0.10)	0.03 (0.05)	0.05 (0.08)	0.03 (0.06)
	Control	0.16 (0.25)	0.42 (0.36)	0.33 (0.32)	0.10 (0.09)	0.05 (0.06)	0.03 (0.04)	0.03 (0.04)
1F2B	Pilot	0.29 (0.22)	0.53 (0.27)	0.41 (0.19)	0.29 (0.15)	0.23 (0.16)	0.16 (0.12)	0.09 (0.07)
	Control	0.41 (0.29)	0.64 (0.28)	0.58 (0.24)	0.45 (0.26)	0.36 (0.26)	0.25 (0.21)	0.19 (0.22)
2F1B	Pilot	0.24 (0.29)	0.53 (0.32)	0.45 (0.31)	0.20 (0.25)	0.11 (0.15)	0.07 (0.07)	0.06 (0.08)
	Control	0.46 (0.40)	0.96 (0.07)	0.94 (0.08)	0.40 (0.25)	0.25 (0.21)	0.12 (0.14)	0.09 (0.12)

*Note*. 1F = one flash; 1F1B = one flash with one beep; 2F = two flashes; 2F2B = two flashes with two beeps; 1F2B = one flash with two beeps; 2F1B = two flashes with one beep.

**Figure 2.**
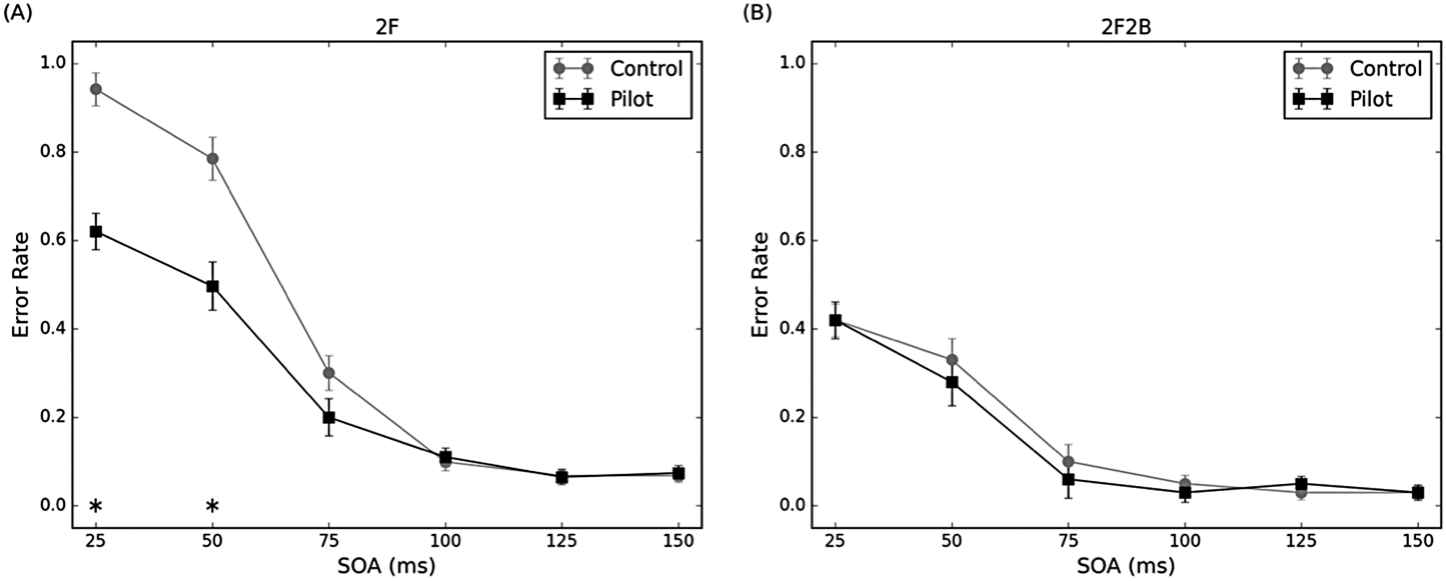
The mean error rate in the (A) 2F condition and (B) 2F2B condition as a function of SOAs for pilot and control groups. Error bars represent ± *SE*, *p < .05. 2F = two flashes; 2F2B = two flashes with two beeps; SOA = stimulus onset asynchrony.

For the 2F condition, the error rate was entered into a 6 × 2 factors ANOVA, with the within-subjects factor of SOAs (25, 50, 75, 100, 125 and 150 ms), and between-subjects factor of groups (pilot, control) (Fig. 2A). The main effect of the group was significant, *F*(1, 64) = 12.55, *p* < .001, *η_p_^2^* = 0.16. The error rate for pilot groups was lower than for the control groups. The main effect of SOA was significant, *F*(2.35, 150.24) = 281.39, *p* < .001, *η_p_^2^* = 0.81. The error rate in 25, 50, and 75 ms was significantly different from that in other SOAs. The interaction between the group and SOA was significant, *F*(2.35, 150.24) = 16.48, *p* < .001, *η_p_^2^* = 0.20. The error rate for pilot groups was only significantly lower than control groups in 25 and 50 ms. Pilots were better at discriminating two flashes than controls at shorter SOAs (25 and 50 ms). Beyond these SOAs, both groups demonstrated comparable discrimination performance.

For the 2F2B condition, the same statistical analysis is also conducted. The main effect of the group was not significant, *F*(1, 64) = 0.19, *p* = .67, *η_p_^2^* = 0.01. The main effect of SOA was significant, *F*(5, 320) = 65.03, *p* < .001, *η_p_^2^* = 0.50. The error rate decreases as SOA increases until SOA 75 ms. The interaction between the group and SOA was not significant, *F*(5, 320) = 0.52, *p* = .76, *η_p_^2^* = 0.01. Two groups perform similarly in this condition (Fig. 2B).

**Figure 3.**
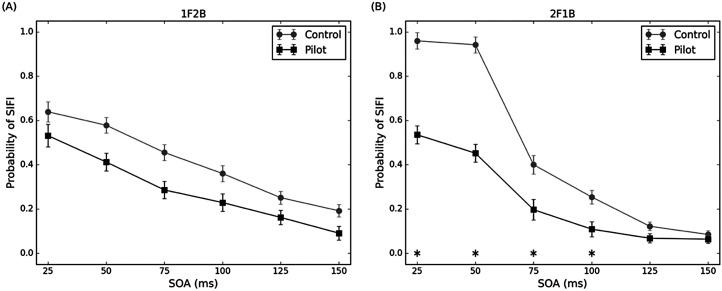
Mean proportion of illusory responses in the (A) fission conditions and (B) fusion conditions as a function of SOAs for pilot and control groups. Error bars represent ± *SE*, *p < .05. SOA = stimulus onset asynchrony.

For the fission illusions (1F2B), the probability of fission was entered into a 6 × 2 factors ANOVA, with the within-subjects factor of SOAs (25, 50, 75, 100, 125 and 150 ms) and the between-subjects factor of groups (Fig. 3A). The main effect of the group was significant, *F*(1, 64) = 7.68, *p* = .007, *η_p_^2^* = 0.11. The probability of fission for pilot groups was lower than for the control groups. The main effect of SOA was significant, *F*(2, 128) = 137.45, *p* < .001, *η_p_^2^* = 0.68. The probability of fission significantly declined as SOA increased. The interaction between the group and SOA was not significant, *F*(1, 128) = 1.34, *p* = .26, *η_p_^2^* = 0.02.

For the fusion illusions (2F1B), the probability of fusion was entered into a 6 × 2 factors ANOVA, with the within-subjects factor of SOAs and the between-subjects factor of groups (Fig. 3B). The main effect of the group was significant, *F*(1, 64) = 49.19, *p* < .001, *η_p_^2^* = 0.43. The probability of fusion for pilot groups was lower than for the control groups. The main effect of SOA was significant, *F*(2.1, 134.11) = 243.59, *p* < .001, *η_p_^2^* = 0.79. The probability of fusion significantly declined as SOA increased. The interaction between the group and SOA was significant, *F*(2.1, 134.11) = 25.32, *p* < .001, *η_p_^2^* = 0.28. The probability of fusion for pilot groups is lower than that for control groups in 25, 50, 75 and 100 ms. Pilots exhibited a lower proneness for SIFI in both fission and fusion conditions than the control group, with a more pronounced advantage in the fusion condition at shorter SOAs.

### Temporal Binding Window (TBW)

**Figure 4.**
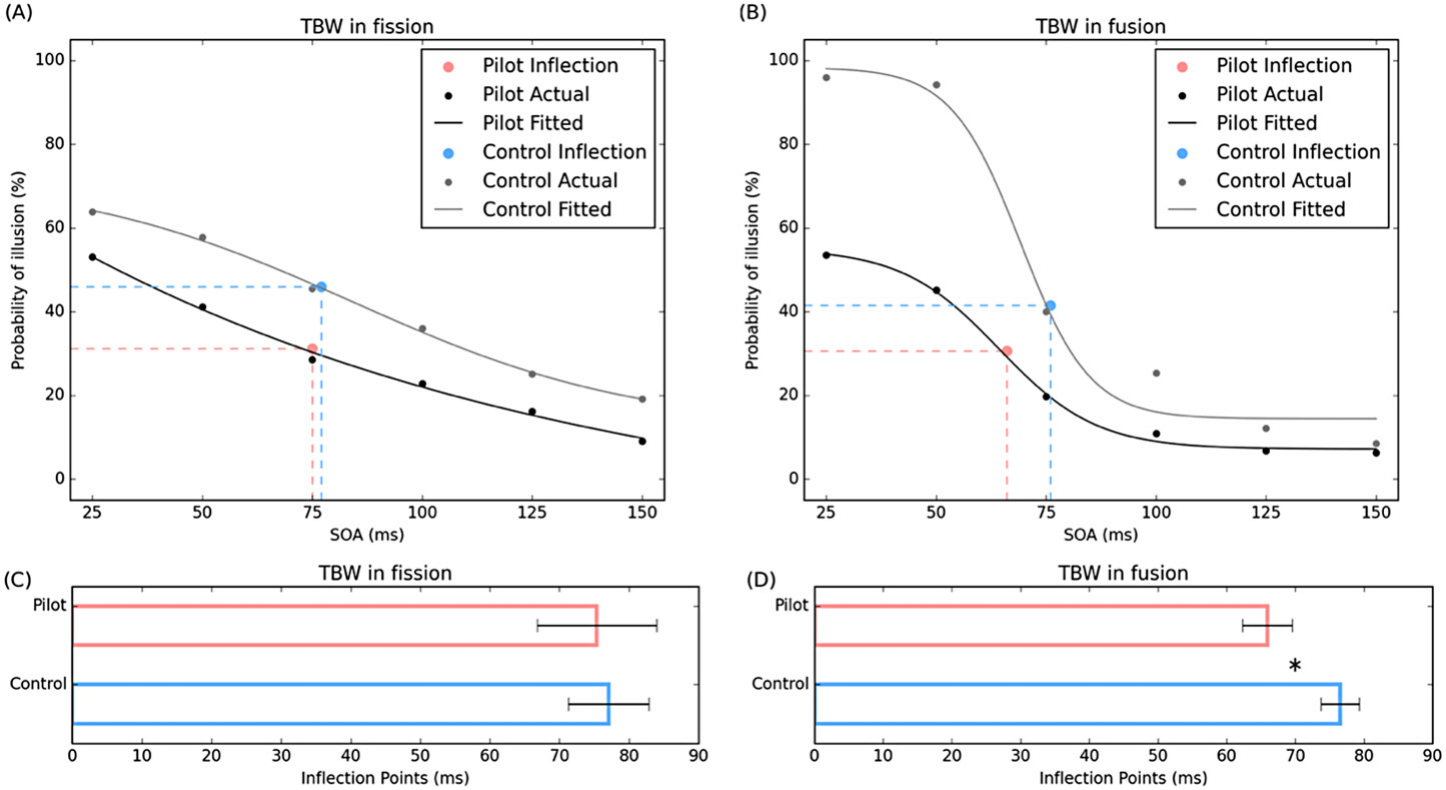
Examples of fitted curves for pilot and control group, and comparisons of the TBW. Sigmoid curves representing the probability of perceiving (A) the fission illusion and (B) the fusion illusion plotted as a function of SOA for pilot group (black curve) and control group (gray curve). For clarity, the curves shown were fitted to the group averaged data, whereas statistical analyses were carried out from individual subject fits. Mean inflection points corresponding to the TBW for all participants in each group are shown in (C) the fission condition and (D) the fusion condition. Error bars represent ± *SE*, *p < .05. TBW = temporal binding window.

For the fission illusions (1F2B), the pilot group showed comparable TBW (*M* = 75.34, *SD* = 42.89) to the control group (*M* = 77.061, *SD* = 33.15), *t*(56) = 0.16, *p* = .87. For the fusion illusions (2F1B), the pilot group showed significantly reduced TBW (*M* = 65.94, *SD* = 18.16) compared to the control group (*M* = 76.539, *SD* = 16.17), *t*(56) = 2.19, *p* = .03. Therefore, pilots exhibited similar TBW of the fission illusion (Fig. 4C) but narrower TBW of the fusion illusion (Fig. 4D) compared to the control group.

### Perceptual Sensitivity and Response Criterion

SDT was employed to determine the factors behind the differing SIFI pattern performances between pilots and controls, distinguishing between shifts in perceptual sensitivity and variations in response criterion (Table 2).

**Table 2.** Mean (*SD*) of SDT measures (*d’*, *ln(β)*) by groups across SOAs in fission and fusion conditions.

Condition	Group	SDT	Overall	25 ms	50 ms	75 ms	100 ms	125 ms	150 ms
Fission	Pilot	*d'*	2.14 (1.42)	0.32 (1.28)	1.10 (1.15)	2.37 (0.78)	2.78 (0.77)	2.90 (0.80)	3.35 (0.69)
		*ln(β)*	−0.88 (0.96)	−0.50 (0.74)	−0.77 (0.83)	−1.30 (0.97)	−1.29 (0.94)	−0.84 (1.02)	−0.57 (0.99)
	Control	*d'*	1.61 (1.42)	−0.09 (0.84)	0.44 (0.84)	1.54 (0.85)	2.12 (0.94)	2.73 (0.79)	2.94 (0.98)
		*ln(β)*	−0.81 (0.89)	−0.33 (0.77)	−0.53 (0.87)	−0.76 (0.86)	−1.05 (0.93)	−1.24 (0.93)	−0.96 (0.65)
Fusion	Pilot	*d'*	2.61 (1.32)	1.46 (1.26)	1.76 (1.27)	2.73 (1.32)	3.12 (1.00)	3.27 (0.83)	3.31 (0.9)
		*ln(β)*	−0.38 (1.18)	−0.86 (1.15)	−0.91 (1.19)	−0.44 (0.86)	−0.13 (1.30)	0.00 (1.20)	0.08 (1.05)
	Control	*d'*	1.88 (1.59)	−0.03 (0.49)	0.11 (0.50)	2.05 (0.96)	2.60 (0.91)	3.18 (0.92)	3.88 (0.88)
		*ln(β)*	−0.67 (1.07)	−0.02 (0.78)	−0.23 (0.78)	−1.45 (0.92)	−1.26 (0.97)	−0.65 (1.12)	−0.40 (1.06)

*Note*. SDT = signal detection theory; SOA = stimulus onset asynchrony.

**Figure 5.**
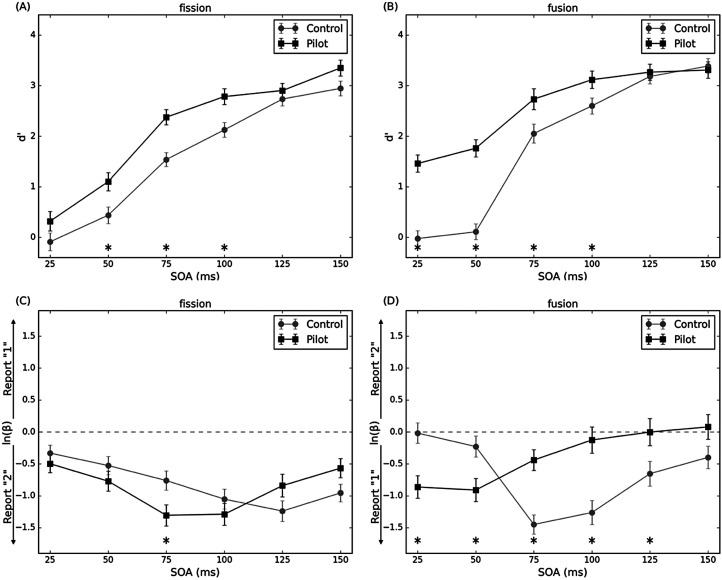
Mean estimates of perceptual sensitivity *d’* and response criterion *ln(β)* as a function of SOAs for pilot and control groups in fission and fusion conditions. (A) In the fission condition, pilots have a general high *d’* across all SOAs. (B) In the fusion condition, pilots have a significantly higher *d’* in shorter SOAs particularly. (C) In the fission condition, pilots have a comparable level of response criterion with control groups that tend to respond two-flash. (D) In the fusion condition, pilots have a significantly different response criterion with control groups. Error bars represent ± *SE*, *p < .05. SOA = stimulus onset asynchrony.

For the fission condition, the calculated *d’* was entered into a 6 × 2 factors mixed ANOVA, with the within-subjects factor of SOAs (25, 50, 75, 100, 125 and 150 ms) and the between-subjects factor of groups (Fig. 5A). The main effect of the group was significant, *F*(1, 64) = 9.64, *p* = .003, *η_p_^2^* = 0.13. The *d’* for the pilot group was higher than for the control group. The main effect of SOA was significant, *F*(2.258, 144.514) = 220.52, *p* < .001, *η_p_^2^* = 0.78. The *d’* significantly increased as SOA increased. The interaction between the group and SOA was not significant, *F*(5, 320) = 2.24, *p* = .10, *η_p_^2^* = 0.03.

The calculated *ln(β)* was entered into 6 × 2 factors mixed ANOVA, with the within-subjects factor of SOAs and the between-subjects factor of groups (Fig. 5C). The main effect of the group was not significant, *F*(1, 64) = 0.19, *p* = .66. The main effect of SOA was significant, *F*(3.673, 235.0640) = 11.62, *p* < .001, *η_p_^2^* = 0.15. The *ln(β)* significantly decreased until SOA 100 ms and then slightly reversed to increase. The interaction between the group and SOA was significant, *F*(53.673, 235.0640) = 5.11, *p* < .001, *η_p_^2^* = 0.07. The *ln(β)* for the pilot group was significantly lower (farther from 0) than that for the control group only at SOA 75 ms.

For the fusion condition, the calculated *d’* was entered into a 6 × 2 factors mixed ANOVA, with the within-subjects factor of SOAs and the between-subjects factor of groups (Fig. 5B). The main effect of the group was significant, *F*(1, 64) = 13.51, *p* < .001, *η_p_^2^* = 0.17. The *d’* for the pilot groups was higher than for the control group. The main effect of SOA was significant, *F*(2.296, 146.9310) = 259.29, *p* < .001, *η_p_^2^* = 0.80. The *d’* significantly increased as SOA increased. The interaction between the group and SOA was significant, *F*(2.296, 146.93120) = 24.83, *p* < .001, *η_p_^2^* = 0.28. The *d’* for the pilot group was higher than that for the control group at 25, 50, 75 and 100 ms.

The calculated *ln(β)* was entered into 6 × 2 factors mixed ANOVA, with the within-subjects factor of SOAs and the between-subjects factor of groups (Fig. 5D). The main effect of the group was not significant, *F*(1, 64) = 1.82, *p* = .18. The main effect of SOA was significant, *F*(3.400, 217.584) = 13.48, *p* < .001, *η_p_^2^* = 0.17). The *ln(β)* significantly decreased until SOA 75 ms and then slightly reversed to increase. The interaction between the group and SOA was significant, *F*(3.400, 217.5842) = 31.62, *p* < .001, *η_p_^2^* = 0.33. The *ln(β)* for the pilot group was lower (farther from 0) than that for the control group at 25 ms and 50 ms. However, after these SOAs, the *ln(β)* for the control group was lower (farther from 0) than that for the pilot group at 75, 100, and 125 ms.

In summary, perceptual sensitivity predominantly explains why pilots report fewer illusions in the fission condition, while in the fusion condition, both perceptual sensitivity and response criterion contribute to the difference between groups.

## Discussion

The present study used double-flash illusion tasks to investigate whether pilots are less susceptible to SIFI and whether their TBW differs from those of non-pilots. We compared illusion rates in both fusion and fission conditions across six SOAs. Our primary finding was that pilots were indeed less prone to SIFI in both conditions as hypothesized. Additionally, we applied SDT to explore the underlying factors. In the fission condition, pilots’ advantage was driven primarily by higher perceptual (i.e., visual) sensitivity in discriminating two flashes. The response criterion result indicates that additional auditory beeps influenced pilots and non-pilots similarly. This is consistent with the similar TBWs observed between groups in the fission condition. In contrast, in the fusion condition, pilots not only exhibited greater visual sensitivity but also showed a more stable response criterion, which suggests that an extra auditory beep did not affect pilots’ judgments of two close flashes as much as it did in the control group. This aligns with the narrower TBW observed for pilots in the fusion condition, implying a distinct multisensory integration mechanism.

In the following sections, we will discuss potential explanations for these group differences in reporting SIFI considering the differences between fission and fusion.

### Inheriting a Visual Advantage

We first suspect that pilots’ reduced susceptibility to SIFI is correlated to their visual advantage. Pilots have demonstrated superior visual processing in many studies (Dror et al., 1993; Ledegang & Groen, 2018; Liang et al., 2024; Shao et al., 2021; Sterkin et al., 2018; Yu et al., 2016). Also, according to the *d′* measure, pilots exhibited higher visual sensitivity. This unisensory benefit likely stems from a specialized training environment that may also reshape how they process multisensory stimuli. This account is supported by between-group comparisons in other expert populations. For example, professional musicians (Bidelman, 2016) and bilinguals (Bidelman & Heath, 2019), the groups with documented auditory-modality advantages, also show both reduced SIFI susceptibility and narrower TBW compared to their controls. Similarly, expert video game players (Di Luzio et al., 2021; Donohue et al., 2010), who are believed to have enhanced visual processing, also demonstrate reduced illusion effects and narrower TBW.

A possible explanation for the group discrepancy is that repeated and prolonged exposure to audio-visual signals is intrinsic to visual or auditory-dominated training. This specialized training allows for enhanced temporal precision in binding multisensory and sensory-motor information because of the plastic reorganization of neural connections between relevant areas (e.g., different cortices), facilitating increased and expedited interaction between sensory modalities through training for optimal and adaptive coordination of the acquired skill (Bidelman, 2016; Bidelman & Heath, 2019; Di Luzio et al., 2021). However, in the fission condition we found no strong evidence for enhanced multisensory integration from the *ln(β)* measure and the TBW, which showed no group differences. The former suggests each participant's bias to respond to auditory beeps over visual flashes, and the latter denotes the SOA at which illusion proneness significantly decreases. Visual sensitivity may be the primary driver of pilots’ reduced fission-illusion susceptibility.

Accordingly, the alternative explanation is that pilots’ visual superiority may directly account for their reduced fission-illusion susceptibility. The Maximum-Likelihood Estimation (MLE) model proposes that observers integrate multisensory cues to minimize the variance of their final perceptual estimate. When combining information across two modalities, the weight assigned to each source is inversely proportional to its uncertainty, closely approximating an ideal Bayesian observer (Ernst & Banks, 2002). Because pilots have lower uncertainty for visual flashes due to their enhanced visual processing, they assign greater weight to vision and therefore experience fewer fission illusions. Moreover, as SOA increases, the temporal separation between the two beeps grows, reducing auditory uncertainty and thus gradually increasing the weight of the auditory cue. Within our measured TBW (around 75 ms), at which the two stimuli are still integrated, this shift is reflected in our *ln(β)* results: as SOA increases, participants’ response criterion increasingly reports the number of beeps instead of the flash. This pattern not only supports the MLE model account but may also imply that our measured TBW closely approximates the true TBW.

### Differences Between Fission and Fusion

Another notable finding is that pilots exhibited a narrower measured TBW than controls in the fusion condition, which is different from those seen in the fission condition. First, visual sensitivity may still play a role at shorter SOAs (25 and 50 ms), as evidenced by pilots’ higher *d’* and lower error rates in the 2F condition. Although the MLE model may account for the relationship between pilots’ visual sensitivity and reduced fusion-illusion report at those SOAs, it cannot explain why control participants increasingly report “1” flash as SOA increases. In the fusion condition, SOA manipulation adjusts the interval between the two flashes. So, according to the MLE model, longer SOAs should increase certainty about the flashes and thus decrease the weight assigned to auditory beeps. Nevertheless, the *ln(β)* measure showed that control participants become increasingly biased toward responding to the beep number (i.e., “1”) as SOA increases from 25 to 75 ms. In addition, both groups performed similarly in the 2F2B condition, which indicates that visual sensitivity alone is insufficient to explain the two groups’ different performances in reporting the fusion illusions. Taken together, it is suggested that pilots and controls differ not only in visual sensitivity but also in their multisensory integration mechanisms (i.e., their TBW) and that both factors jointly contribute to the reduced fusion-illusion susceptibility in pilots.

An interesting question is why pilots’ performance diverges between fission and fusion conditions relative to controls. A potential explanation for this difference involves temporal ventriloquism, where the auditory stimulus alters the perception of the timing between the visual stimuli, making them appear more separated or closer together, contingent on the SOA (Narinesingh et al., 2017). Notably, this effect only occurs within the known TBW for multisensory integration (Morein-Zamir et al., 2003), which could explain why pilots exhibit a “gentler slope” in their response trend at the shorter SOAs.

In addition, many previous studies have reported generally stronger fission effects compared to fusion when set against unimodal flash conditions (McGovern et al., 2014; Narinesingh et al., 2017; Vanes et al., 2016). Different neural mechanisms are proposed to explain these illusions, as indicated by EEG and fMRI research (Mishra et al., 2007, 2008; Watkins et al., 2006, 2007). For instance, the fission illusion appears to be processed more swiftly, with ERP analyses suggesting that the latencies of major components associated with fission occur at 110, 120, and 130 ms, reflecting activations in the auditory, visual, and superior temporal cortices, respectively (Mishra et al., 2007). Conversely, the major components of the fusion illusion are detected at later latencies (180 and 240 ms) but also involve interaction from the superior temporal cortex to the visual cortex (Mishra et al., 2008). This delayed processing time for the fusion illusion may account for the higher susceptibility to fusion at lower SOAs compared to fission, as observed in our study. Consequently, pilots’ superior performance in the fusion condition could be due to the accelerated processing of multisensory information. This enhanced processing speed is particularly evident in the shorter SOAs in the fusion condition.

However, a key limitation and important unsolved question is whether pilots’ lower susceptibility to the SIFI reflects pre-existing perceptual skills that lead them more likely to apply for and be selected into pilot training, or whether it arises from perceptual learning through flight experience (Quinn et al., 2023). Pilot selection is notably stringent and involves rigorous assessments of visual sensitivity, attentional allocation, and visual acuity, so it is plausible that our pilot sample represents a subpopulation with inherently superior multisensory temporal processing. Therefore, the primary contribution of this study is to document and analyze this group difference in SIFI susceptibility and to motivate future research aimed at disentangling innate ability from experience-driven plasticity.

Another limitation is the predominantly male makeup of our participants, which could limit the broader applicability of our results. Future research should include a more diverse group of participants to enhance the generalizability of the findings.

## Conclusion

Our findings indicate that pilots are less susceptible to the sound-induced flash illusion in both fission and fusion conditions, yet they exhibit a narrower temporal binding window (TBW) only in the fusion condition. Two alternative explanations have been discussed for this group difference, suggesting distinct multisensory integration mechanisms underlying the fission and fusion illusions.
